# A multidimensional strategy to managing dysfunctional breathing and exercise-induced laryngeal obstruction in adolescent athletes

**DOI:** 10.1186/s13102-023-00804-2

**Published:** 2024-01-11

**Authors:** Liv-Jorunn Kolnes, Trine Stensrud, Oddbjørn Klomsten Andersen

**Affiliations:** 1 Faculty of Health, VID Specialized University, Diakonveien 12-18, 0370 Oslo, Norway; 2 Department of Sports Medicine, Norwegian School of Sport Sciences, Oslo, Norway

**Keywords:** EILO, Adolescent athletes, Thoracic versus diaphragmatic breathing, Postural alignment, Physiotherapy intervention, Cognitive behavioural therapy, Rehabilitation plan

## Abstract

**Background:**

Exercise induced laryngeal obstruction (EILO) causes inspiratory distress in the upper airway in many adolescent athletes. The nature of EILO is not fully understood, and effective management strategies are lacking. This study aimed to assess the effectiveness of a multidimensional individually tailored intervention, including Norwegian Psychomotor Physiotherapy (NPMP), elements of cognitive behavioural therapy and a rehabilitation plan, in reducing inspiratory distress and dysfunctional breathing in adolescent athletes with EILO.

**Methods:**

A mixed methods design, which combined qualitative and quantitative research, was used. Data, including subjective experiences of respiratory distress, findings from body examinations and objective measurements of lung function and aerobic capacity were gathered before and after a five month intervention involving 18 participants.

**Results:**

Following the intervention, the participants showed a reduction in respiratory distress and anxiousness associated with their breathing difficulties. Furthermore, the participants reported to be more in control of their breathing. The body assessments revealed a more functional breathing motion and improved posture, which imply that the breathing was less thoracic and more diaphragmatic in rest and exercise in all participants after the intervention.

**Conclusions:**

Our results suggest that a multidimensional individually tailored intervention, including NPMP based physiotherapy, cognitive behavioural therapy elements, and a rehabilitation plan may reduce inspiratory distress and dysfunctional breathing in athletes with EILO.

**Trial registration:**

ClinicalTrials.gov Protocol Registration and Results system NCT06033755, date of registration: September12, 2023. Retrospectively registered.

## Introduction

Exercise induced laryngeal obstruction (EILO) has been reported to cause inspiratory distress in the upper airway in many adolescent athletes. EILO is a subcategory of inducible laryngeal obstruction (ILO) occurring in the course of the inspiratory phase of respiration and physical exertion is the trigger provoking transient supraglottic and/or glottic narrowing. EILO likely interferes with participation in sports, performance and quality-of-life in athletes [1, 2]. Available evidence indicates prevalence of 5 – 10% in the general adolescent population [3, 4], with higher rates in adolescent athletes [5]. The nature and underpinnings of EILO are not fully understood, and effective management strategies are lacking. Even though symptoms of EILO are typically located in the laryngeal area, EILO is often mixed with asthma and treated with anti-asthmatic medication, despite a lack of evidence to support an asthma diagnosis, and lack of response to the medication [5–8].

Theories put forward to explain EILO include psychological causes [9, 10], laryngeal hypersensitivity [11, 12], increased autonomic activity and exposure to high levels of stress [13–16]. Increased activation of intrinsic laryngeal muscles and the trapezius muscle following whole body autonomic nervous system (ANS) activation has been demonstrated in females indicating that laryngeal muscles, as well as other muscles involved in the respiration, react in response to ANS activation [17]. Stress reactivity in individuals with EILO may also reflect dispositions to stress responses recognized in competitive young athletes in general [16]. More recently, a pattern of dysfunctional breathing has been suggested to be involved in EILO [1, 15, 18]. Dysfunctional breathing is a term describing changes in the normal biomechanical patterns of breathing resulting in temporary or chronic symptoms such as dyspnoea and respiratory distress [19, 20]. When the normal relaxed respiratory cycle is replaced with patterns of abnormal breathing, a number of patterns have been identified including hyperventilation syndrome, periodic deep sighing, forced abdominal expiration and a dominant thoracic breathing [21]. Thoracic breathing has lately been recognized in many adolescent athletes diagnosed with EILO [15, 22].

There is a lack of systematic investigations of treatment strategies for EILO. The literature indicates that strategies that can improve respiratory symptoms involve laryngeal control therapy and respiratory retraining techniques [23], inspiratory muscle training [24], physiotherapy [1, 15] and speech and language therapy [25, 26]. With the exception of physiotherapy, major features in these treatments are their focus on breathing techniques and the inspiratory phase of the respiration. Surgery is endorsed for a selected group of patients with moderate to severe supraglottic obstruction [2, 27]. Release of breathing, re-establishing a balanced posture and reducing tension in structures directly or indirectly associated with the larynx, are addressed in physiotherapy [1, 15] and in some speech and language therapies [28, 29]. For instance, the Norwegian Psychomotor Physiotherapy (NPMP) approach, which aims to promote diaphragmatic and functional breathing where inspiration can take place without restrictions, was implemented in a case study involving four adolescent athletes diagnosed with EILO and who displayed a pattern of thoracic breathing [15]. The participants displayed less subjective respiratory distress, more functional breathing, reduced inappropriate laryngeal movements during maximal exercise, and improved lung function following a five months physiotherapy intervention combined with features of cognitive behavioural therapy and modifications within the training schedules [15].

The aforementioned study holds promise, however, there is a need for studies on therapeutic interventions on larger numbers of adolescent athletes with EILO. Thus, the overall aim of this study was to examine whether an intervention consisting of NPMP based physiotherapy, elements of cognitive behavioural therapy and an individually tailored rehabilitation plan can reduce inspiratory distress and dysfunctional breathing in a higher number of athletes presenting with EILO.

## Methods

### Design

A mixed methods design, which combined qualitative and quantitative data was used in the present study. The data were gathered before and after a five months intervention period in the eighteen participants. The qualitative material is descriptive and consists of a) recordings of subjective experiences of respiratory distress explored via a questionnaire also used by Roksund [30], and b) qualitative descriptions of the findings of the body examinations. Quantitative measurements include objective measures of lung function and aerobic capacity. Each participant had between eight and twelve physiotherapeutic consultations during the intervention.

### Participants

Inclusion criteria were adolescent athletes with symptoms of EILO and who were referred to NPMP treatment. Twenty two adolescent athletes between 13 – 19 years who were referred to NPMP treatment due to shortness of breath and possibly EILO between December 2018 and August 2022 were asked by the physiotherapist to take part in the study. Eighteen of these athletes (17 females, 1 male) were included. Before inclusion, they were subjected to an introductory interview based on the questionnaire developed by Roksund [30] to determine whether their symptoms were indicative of EILO. Exclusion criteria were additional diagnosis or respiratory symptoms not adhering with EILO. Four athletes referred to NPMP due to possible EILO were not included due to additional diagnosis.

The participants were referred by doctors or paediatrics working with respiratory problems in children and adolescents. Laryngeal movements had been measured using a continuous laryngoscopy exercise test (CLE) [31, 32] to verify the condition in four participants. Subjective experiences of inspiratory distress located in the larynx area during physical activity and recordings in the questionnaire confirmed the EILO condition in all participants.

### Recordings of respiratory distress

Subjective experiences of respiratory distress were explored pre and post intervention through a comphrehensive history taking and in the interview based on the questionnaire previously used by Roksund to obtain the medical history, level of engagement in sport, information of symptoms related to exercise, activity level of the subjects and the influence of the symptoms on daily life [30]. A visual analogue scale served to grade the severity of symptoms (see Table 2).

### The Body examination

The intervention was based on a thorough body examination commonly used in NPMP in which bodily imbalances in general and restrictions associated with the breathing in particular were assessed. The observations conducted by the physiotherapist during the body examinations were assessed and noted qualitatively and descriptively. They were not graded, since we did not use examination methods that grades the findings, but assessed and descriptively described the respiration, posture, muscular tension and functioning, the latter indicating bodily flexibility and ability to relax.

### Measure of lung function and cardiac respiratory fitness

Lung function was measured by maximal expiratory flow-volume curves and ventilatory capacity by maximal voluntary ventilation (MVV) (MasterScreen Pneumo spirometer; CareFusion, Hochberg, Germany) according to current guidelines [33]. Lung function is presented as forced vital capacity (FVC) and forced expiratory volume in 1 s (FEV_1_). Lung function was measured before and 1, 3, 6 and 10 min after exercise test and a reduction of ≥ 10% in FEV_1_ was defined as exercise induced bronchial constriction (EIB).

Exercise test. Maximal oxygen consumption (VO_2max_) was measured using a mixing-chamber gas analysis system (OxyconPro analyzer; Jaeger, Würtzburg, Germany) on a treadmill (ELG 90/200 Sports; Woodway, Weil am Rhein, Germany). Prior to the test, the participants performed a 10 min standardized warm-up, tailored to the individual. Output speed was adjusted to the participant’s fitness level, and the speed was thereafter increased by 1 km·h^−2^ every minute until voluntary exhaustion. The incline was set to 5% and did not change during the test. VO_2max_ was defined as the mean of the two highest 30 s measurements coupled with a maximal respiratory exchange ratio (RER_max_) > 1.1. The following variables were extracted and included in the analyses; VO_2max,_ maximal minute ventilation (VE_max_), maximal breathing frequency (BF_max_), RER_max_, maximal heart rate (HR_max_), time to exhaustion and Borg scale for self-perceived exhaustion. Breathing reserve (BR) (%) was estimated as MVV-VEmax/100 × 100.

### The intervention

The intervention had a multidimensional approach that included physiotherapy, elements of cognitive behavioural therapy and an individually tailored rehabilitation plan, all handled by the first author (who has a PhD in sports science and is a certified NPMP specialist) in close collaboration with the participants and their parents.

#### A: The physiotherapy intervention

Was based on the main principles of NPMP and addressed bodily and respiratory restrictions identified in the body examinations. Intervention details were recorded during each consultation. The NPMP approach differs from traditional physiotherapy in that it understands the body to be a functionally integrated entity, inferring that constriction in one part of the body influences the entire body [34]. A key idea is that unresolved and difficult feelings, as well as continuing and high levels of life stress, are reflected in the body, including in the ANS, which is significant in this context as it relays experiences of stress to the respiratory system typically resulting in constricted breathing [35]. By the use of massage, stretching and grounding exercises, the aim is to release bodily tension and restricted breathing to increase bodily flexibility and stability, and to help patients tune into the body and understand that stressful experiences, relations and feelings are also bodily. The observing of respiration and changes in its patterns, rhythm and depth, is essential to NPMP. It is assumed that restricted breathing over time will affect the mechanisms of breathing, bodily expressions, functions and movements negatively [36, 37]. A key aim is typically a free and diaphragmatic breathing that moves through the thorax without restrictions. A breathing that is free does not require much thinking since it is controlled by the ANS.

Accordingly, the focus of the physiotherapy was not merely on improving the participants breathing as such, it also addressed bodily dealignments and bodily constraints, as these over time, can affect the function of breathing negatively. The physiotherapy was tailored to each individual participant according to their specific bodily restrictions related to respiration, posture, muscle tension and function. These features closely interact with one another. The restoration of the breathing was approached through careful release over time such that participants could integrate potential changes in their daily breathing. Several NPMP measures and practices were applied to facilitate and enable diaphragmatic respiration, including these:

- A balanced standing position, where the body is aligned and neck and shoulders are in a neutral position.
- A careful pressure and/or vibrations on the middle thoracic cage during the expiratory phase.
- Careful movement and retraction of larynx by the therapist.
- Massaging and stretching muscles directly related to, or assisting in, the breathing, such as muscles of the upper chest and neck.
- Massage and/or stretching of tense muscles in other body parts.
- Expiratory pursed lip practices can incite diaphragmatic breathing.
- Enacting a prolonged sigh through the mouth during the expiratory phase, where the mouth and jaws are relaxed, and wait for a “respiratory response”, was practiced and repeated in each consultation with the participants. The procedure may be labelled the “expiratory breathing practice” or “*relaxed breathing out practice*” (R-BOP). We prefer using “practice” to avoid an unfortunate focus on performing a right “technique”. R-BOP may be accompanied by holding one or both hands on the upper abdomen, just below the sternum, and wait for motion in the abdomen that accompany diaphragmatic inspiration.

##### B: Elements of cognitive-behavioural therapy

Cognitive behavioural therapy (CBT) involves a number of talking approaches and techniques adressing thoughts, emotions and behavior. It is commonly used to treat mild to moderate anxiety and depression, but is also useful for other mental and physical health issues. Key features of CBT are its focus on identifying and understanding existing ways of thinking and behaving, and on equipping people with tools to change their cognitive and behavioral patterns [38].

In this study, during the physiotherapy consultations with the participants, their thoughts and behaviour about training and competing were in particular addressed. To avoid respiratory symptoms and improve breathing during training and competition, the participants were provided with measures, such as holding one or two hands on the abdomen to cheque that the breathing movement was downwards, or diaphragmatic, as opposed to an upward movement taking place in the upper chest. This should be chequed out before the activity started and before further increase of intensity. The ultimate aim was to ensure a breathing that did not trigger EILO symptoms and to find a way that worked for the individual, in which she or he felt to be more in control of her/his breathing. One way to acquire such breathing was initially during low intensity training. Also, it was essential that the participants were aware of the position of the neck and kept the neck in a neutral position, since this is the ideal position for the airflow and laryngeal movements.

In addition, to lower the pressure and expectation about producing top results, which is a potential stressor that frequently trigger an upwards breathing movement (i.e., thoracic breathing), the participants were advised not to focus on results when they started to compete but rather on doing their sport technically well and think of it as a “routine training”. Lastly, to reduce overall stress and increase time and space for rest and restitution, the participants were encouraged to identify situations and activities (e.g., social media) in their daily life could be altered in terms of their level of involvement.

#### C: Rehabilitation plan

An individually tailored rehabilitation plan is required for the rehabilitation process of athletes recovering from acute injuries or chronic overuse of musculoskeletal structures and/or functions. Such a plan needs to take into account that a frequent goal of the person is to return to the same activity as before the injury occurred or the overuse materialized [39]. Since EILO primarily occurs during high-intensity exercise, usually lasts for months to years, and is often associated with thoracic breathing involving compensatory changes (e.g., increased activation of respiratory muscles and postural dealignments of the neck and shoulders) [40], it appeared appropriate to approach rehabilitation within the principles of a long-lasting overuse condition.

The rehabilitation plan for the participants in this study had a functional take and involved activities and exercises required for the athletes to return to their specific sports. Limiting further impairments and maintaining aerobic capacity without intensifying the EILO condition were key to the rehabilitation. The rehabilitation plan was individually tailored, but commonly included these three phases:

##### Phase 1 (week 1 – 6)

During the first intervention phase the participants were instructed to avoid activities that could lead to thoracic breathing which would stress respiratory muscles and laryngeal structures. The primary focus of the training was to take part in exercises with low intensity, while at the same time ensuring a diaphragmatic breathing.

Low intensity training implied that the participants could participate in large parts of their usual training activities, such as technique and strategic features where aerobic intensity is usually low. To help arrive at a diaphragmatic breathing, the participants were encouraged to use the R-BOP procedure (as described in the physiotherapy intervention), before and during natural pauses of the training. At the same time, postural alignment issues were addressed. For instance, given that the ideal position of the cervical spine for laryngeal movements is a neutral position, participants were encouraged to focus on keeping the shoulders in a lower position and the neck in a neutral position. Lastly, good warm up to ensure diaphragmatic breathing and stretching tight muscles were emphasized in all phases.

##### Phase 2 (week 7 – 12)

As tension in the laryngeal area and in respiratory muscles decreased and the breathing became more diaphragmatic both at rest and during training, the participants were advised to gradually increase the intensity of training to moderate aerobic training intensities (e.g., 72–87% of HRmax). It was still important to avoid activities and intensities that would trigger high costal breathing. The possibility of using the R-BOP was emphasized throughout the remaining phases to ensure a diaphragmatic breathing before all activities, but also during daily life activities.

##### Phase 3 (week 13 – 20)

As a diaphragmatic breathing pattern became more regular, the participants could gradually perform higher intensity training (e.g., 87–100% of HRmax) and begin competing in their respective sports. The basic goal for taking part in competitions was that the body functioned and breathing was diaphragmatic, rather than focusing on achieving top results or playing whole matches. Team athletes could, for instance, play parts of the game to ensure a functional breathing and feeling in control of the breathing before playing whole games. This was also a period when diaphragmatic breathing became more stabilized, a continuing process.

### Statistics

Descriptive data are provided as median and interquartile range for continuous variables and counts for categorical variables. Continuous variables were normally distributed and a paired sample t-test with Bonferroni correction was performed for assessment of changes in physiological variables and in self-reported symptoms from pre to post intervention. In addition, we checked the results with a non-parametric test and the analysis showed almost equal *p*-values for the variables in Tables 2 and 3. Results are expressed as mean values with 95% confidence intervals (CI). *P*-values ≤ 0.05 were considered statistically significant.

## Results

### Descriptive data

All participants reported respiratory problems indicative of EILO at high intensity training at baseline. The participants were competing in the following sports (bandy, cross-country skiing, handball, horse-riding, soccer, speed skating, and volleyball), with some taking part in two sports. The participants reported their first experience of EILO symptoms between 1 and 8 years (mean 3.2 years) prior to inclusion and were all interested to receive treatment and take part in the study. None of the participants had a doctor-diagnosed asthma during the intervention period. Eleven had previously used anti-asthmatic medication. Only three reported improvement in respiratory symptoms by using this medication, while the remaining reported no effect. Further descriptive characteristics are presented in Table 1.

**Table 1:** Characteristics of the 18 participants at baseline. Results are given as median and interquartile range for age, weight and height and number for doctor diagnosed asthma, allergy, breathing problems, training volume and competition level

Variables	Participants (*n* = 17 ♀ and one ♂)
Age (years)	15.0 (4)
Height (cm)*	172 (8)
Weight (kg)*	61.4 (14)
Dr. diagnosed Asthma ever n	4
Dr. diagnosed Allergy ever n	7
Breathing problems n	18
Training volume (hours/week)	
≥ 10 h	3
7–9 h	8
4–6 h	7
Competition level	
International	1
National	7
Regional	7
Local	3

^*^n = 17 females and one male, Dr. diagnosed = doctor diagnosed

### A: Respiratory distress

#### Pre-intervention

All participants reported to have EILO symptoms during high intensity activity (i.e., hard training and competition) prior to the intervention. Additionally, seven participants had symptoms during activity with moderate intensity (i.e., jogging) and two participants reported symptoms during all intensities, including low intensity activity (i.e., fast walking/slow jogging). Assertions such as “The problem occurs during physical exertion”, “I have problem(s) inhaling”, “I feel tightness in the throat”, and “The problem ends quickly when the activity stops” were endorsed by all participants at baseline. Occurrence of wheezing at baseline was reported in fourteen participants. Prior to the intervention, anxiousness about the symptoms was reported in ten participants and the feeling of having no or little control of the symptoms was reported in twelve participants. Stress was stated to be a trigger for EILO symptoms in twelve participants, while two participants reported extremely hot weather and one person considered cold weather to be a trigger.

### Post-intervention

Eighteen participants completed the five months intervention period and reported improvements on all measures explored through the interview based on the Røksund questionnaire [30]. All participants reported that they felt the breathing had improved during the course of the intervention and that symptoms of EILO occurred less frequent compared to the baseline situation. Six participants felt *some improvement*, and twelve felt *considerable improvement*, among which four participants indicated that they had *fully recovered*. Fourteen participants reported to still have EILO symptoms during high intensity activity, two participants had symptoms during activities of moderate intensity and one participant had symptoms during low intensity activities. However, these episodes were reported to be of reduced intensity compared to the baseline measurements, as shown in Table 2. In addition, anxiousness connected to the problem of breathing had lessened (*p* ≤ 0.01), and feelings of being in more control of the breathing had increased (*p* ≤ 0.001). Lastly, the occurrence of abnormal sounds, or wheezing, were significantly reduced (*p* ≤ 0.01).

**Table 2:** Reported respiratory exercise related symptoms pre and post intervention on a scale from 1–5: 1 = never; 2 = sometimes; 3 = often; 4 = regularly; 5 = always. Results are given as mean and 95% confidence interval (CI) (*n* = 18)

Statements/score Q	Mean (95%CI) Pre intervention	Mean (95%CI) Post intervention	*p*
I have breathing problems during low intensity training	1.1 (0.9, 1.3)	1.0 (1.0, 1.0)	ns
I have breathing problems during high intensity training or sport competitions	3.8 (3.2, 4.4)	2.4 (1.8, 3.0)	≤ 0.01
The breathing problems are worse during competitions compared to high intensity training	2.7 (1.8, 3.6)	2.8 (2.1, 3.5)	ns
I feel tightness in my chest	1.7 (1.2, 2.2)	1.4 (0.8, 2.0)	ns
I become dizzy/nauseous and feel I am fainting	1.5 (1.2, 1.8)	1.3 (0.9, 1.6)	ns
I can hear abnormal sound / wheezing	3.5 (2.7, 4.4)	1.9 (1.3, 2.4)	≤ 0.01
I become anxious when breathing problems occur	1.7 (1.3, 2.1)	1.2 (1.0, 1.4)	0.01
I stop doing high-intensive training due to the breathing problems	2.1 (1.7, 2.6)	1.7 (1.2, 2.2)	ns
I can control the breathing problems when they occur	1.9 (1.3, 2.5)	3.9 (3.4, 4.5)	≤ 0.001

### B: Body examination

#### Pre-intervention

The body assessment revealed high levels of bodily restriction, such as constricted and thoracic breathing, raised muscular tension, postural stiffness and dealignments. The body examination included these findings pre-intervention:

- **Respiration:** A constricted breathing was identified in all participants before the intervention. The characteristics of the constriction varied and primarily included upper chest breathing, suppressed expiratory phase, and reduced respiratory depth and movement.
- **Muscular:** Raised muscular tension was revealed in a number of muscles in all participants, in particular in muscles involved in respiration, but also in muscles of the back and the lower limbs.
- **Posture:** Diverse postural dealignments were detected in most participants, including elevated shoulders, a forward head posture, hyperextension of the neck, and increased forward pelvic tilt along with thoracic stiffness and a stiff standing position with hyperextended knee joints.
- **Functioning:** Reduced ability to relax and to let go during passive movements of under extremities and/or the neck were recognized in five participants. This is frequently accompanying increased whole body tension.

These findings revealed that the diverse bodily restrictions conveyed in the participants extended beyond laryngeal structures. Along with the participants´ EILO associated symptoms, the findings indicate a range of potential limitations and mechanical constraints which, over time, impede a functional respiration both in rest and during activities with increased ventilatory demand.

### Post-intervention

The body examination involves these findings by the end of the intervention:

- **Respiration:** The respiration was less constricted and more diaphragmatic in all participants. The breathing movement was downward, involving synchronized motion of the entire rib cage and abdomen, and respiratory depth was more satisfactory.
- **Muscular:** The level of tension in muscles involved in the respiration was reduced in all participants, but these muscles were still moderately tense in many participants (n = 13). Normalized tension in respiratory muscles was observed in the five remaining participants.
- **Posture:** Posture was more aligned (i.e., in particular the neck) and standing position appeared more flexible in all participants. Even so, those who had a tendency towards hyperextending their neck during rest and/or high intensity activity reported a continued focus on keeping the neck in a neutral position.
- **Functioning** had improved*,* referring to the ability to relax and let go during passive movements of the neck and/or extremities, was recognized in the five participants who had reduced such ability before the intervention.

Accordingly, the body examinations post intervention showed a more functional and diaphragmatic respiration, less tension in respiratory muscles, and improved postural alignments in the participants. The athletes reported that they had become more aware of their breathing and had learned to adjust the intensity of physical activities to avoid respiratory distress to occur. If and when they sensed any respiratory symptoms, they could reduce the intensity of the activity slightly in order to continue the current activity. Being able to complete the activity appeared to provide a sense of self confidence concerning the potential of returning to the actual sport in good time. The participants reported that the relaxed breathing out practice (R-BOP), an effective practice facilitating a diaphragmatic breathing, was extremely accessible and helpful and was used regularly, before and during physical activities, as well as during daily life situations to obtain a deeper breathing.

### C: Lung function and exercise test

One participant was not subjected to lung function and exercise testing due to technical treadmill problems, but completed the intervention and the pre- and post-interviews exploring subjective experiences of respiratory distress. Overall, no changes in the physiological variables or time to exhaustion were observed for the whole group and none of the participants developed exercise induced bronchial constriction (EIB)) during the pre and post exercise tests (Table 3).

**Table 3:** Lung function physiological variables during maximal exercise test, time to exhaustion and Borg scale score immediately after the exercise test pre and post intervention in all participants. Results are presented as mean and 95% confidence intervals (CI) (*n* = 17).

Variables	Mean (95%CI) Pre intervention	Mean (95%CI) Post intervention	*p*
FVC (L)	4.1 (3.7, 4.6)	4.2 (3.8, 4.7)	.125
FEV_1_ (L)	3.6 (3.2, 4.0)	3.6 (3.2, 4.1)	.312
MVV (L·min^−1^)	120.0 (104.6, 130.7))	122.1 (107.5, 135.8)	.463
Ve_max_ (L·min^−1^)	120.3 (102.5, 145.0)	121.4 (104.5, 144.2)	.602
BR (%)	-1.4 (-15.1, 5.3)	-0.7 (-10.8, 6.4)	.764
VO_2max_ (ml·kg^−1^ ·min^−1^)	51.9 (47.0, 58.9)	51.6 (45.3, 57.9)	.794
RER_max_	1.14 (1.08, 1.18)	1.14 (1.11, 1.18)	.788
HR_max_	199 (194, 203)	198 (192, 203)	.292
Borg scale	18.6 (17.9, 19.3)	18.5 (17.7, 19.4)	.718
Time to exhaustion (seconds)	541.7 (486.8, 607.9)	532.8 (482.8, 619.9)	.520

*FVC* Forced vital capacity, *FEV1* Forced expiratory volume in one second, *MVV* Maximal voluntary ventilation, *Ve* Maximal minute ventilation, *BR* Breathing reserve, *VO*_*2max*_ Maximal oxygen uptake, *RER*_*max*_ Maximal respiratory exchange rate, *HR*_*max*_ Maximal heart rate

Looking closer at the four participants who reported no respiratory symptoms after the intervention, no consistent changes occurred in the physiological results.

## Discussion

The results demonstrate that a multidimensional intervention consisting of physiotherapy which addresses diaphragmatic breathing, elements of cognitive behavioural therapy and a tailored rehabilitation plan might reduce symptoms of inspiratory distress in adolescent athletes presenting with EILO. The reduction of symptoms could not be explained by physiological improvements in lung function. The findings support the findings in previous work on physiotherapeutic interventions addressing diaphragmatic breathing in individuals with EILO [1, 15, 41].

A key finding in all participants at baseline was dysfunctional breathing (i.e., thoracic breathing), increased tension in respiratory muscles and forward head posture. While a neutral or lower vertical position has been established as the optimal position for laryngeal movements, thoracic breathing and forward head posture likely generate an elevated position of the larynx within the neck and paradoxical forces within the laryngeal tract [42, 43]. Diaphragmatic breathing implies that during inhalation, the diaphragm, which is the main muscle of inspiration, contracts and descends such that a downward tracheobronchial pull is exerted in order to move the larynx into the position necessary for optimal airflow [28, 44]. As opposed to diaphragmatic breathing that involves abdominal motion and synchronized motion of the upper and lower rib cage and abdomen, thoracic breathing involves greater upper chest motion, produced by the accessory muscles of inspiration (e.g., external intercostals, sternocleidomastoid, upper trapezius, and scalene muscles) which typically become increasingly activated during physical exertion [45]. In addition, extrinsic (i.e., infra- and suprahyoid muscles attached to the hyoid bone, of which the larynx is also attached) and intrinsic muscles of the larynx assist in laryngeal movements and in keeping the larynx in a neutral position, are activated along with other accessory breathing muscles [28]. Consequently, thoracic breathing may, over time, affect the mechanisms of breathing and the musculoskeletal system in general, as well as health and performance negatively.

In this present study, participants respiratory muscles were possibly tense due to the reduced, or lack of, diaphragmatic descent and associated compensatory changes within the overall respiratory system, including postural de-alignments (e.g., forward head posture and increased extension of the lower cervical spine) impairing the neuromuscular control and coordination of cervical movements during respiration [46, 47].

Accordingly, and as have been emphasized in other relevant work [1, 23, 41, 48], facilitating diaphragmatic breathing, release of tension in respiratory muscles and improving postural dealignments particularly in the neck, were of key importance in the intervention. These are elements that are strongly connected and which need to be addressed simultaneously since changes in posture affect the breathing and vice versa [49]. Features which might help change the breathing pattern towards diaphragmatic breathing were addressed in all parts of the intervention, including a) the physiotherapy sessions where the breathing was addressed directly but mostly indirectly, b) by addressing the participants thoughts and cognitions about how to be aware of the breathing and adjust training intensity to a level where respiratory symptoms are minimized, and c) through an individually tailored rehabilitation plan. Together, these elements may have fostered a more efficient communication within the respiratory system post intervention resulting from a change in the breathing from being thoracic to being more diaphragmatic and functional, as demonstrated in the participants experiences of improved breathing and reduced symptoms of EILO during exercise.

It can be noted that we did not put any emphasis on nose breathing in this intervention, as have been the case in other studies [1, 23]. Nose breathing may facilitate diaphragmatic breathing by providing resistance to the expiration. Nevertheless, in our experience, using the R-BOP and a prolonged sigh through the mouth during the expiratory phase, where the mouth and jaws are relaxed, and wait for a “respiratory response”, is an easy, available and effective way of facilitating diaphragmatic breathing for most individuals both in rest and during physical exertion.

The intervention approach used in this study reflect what could be labelled a *global* approach, viewing EILO, and the larynx, as connected to the respiratory system and body as a whole, as opposed to a *local* take, which tend to focus on laryngeal structures [22]. It might be theorized that the coordinated function of respiratory muscles and associated structures, and a good postural alignment are key elements in optimizing breathing [43, 47]. Our results support the assumption that thoracic breathing is a key component in dysfunctional breathing and EILO and that breathing pattern analysis and whole body postural alignment issues should be included in assessments of otherwise healthy adolescent athletes with EILO [1, 41, 48]. Our results further suggest that a conservative approach (i.e., therapies addressing the pattern of breathing, including physiotherapy and speech and language therapy) should be advised in the initial investigation and management of otherwise healthy children and adolescents with symptoms suggesting EILO, and that the CLE test may be considered to confirm the condition and characterize potential anatomical abnormalities that contribute to EILO [1]. In addition, in order to explain the condition, increase participants awareness and control of the breathing both in rest and during exercise, educational means, (e.g., elements of cognitive behavioural therapy) that involve the athletes, their parents and/or coaches seem crucial in managing a condition like EILO [1].

Moreover, the results indicate that effects of stress on the body (including on the breathing) should be considered within this area. In the present study, stress was experienced to be a central trigger for EILO symptoms, and anxiousness and feelings of not being in control of the symptoms were reported by many of the participants. The various developmental and transitional challenges that adolescents are faced with, along with sport related stressors, likely produce stress responses in the body of many adolescent athletes [50]. Evidence suggests that persistent stress and anxiousness interacts with the respiration and the larynx via the ANS [17, 51]. It has been hypothesized that high levels of long term physiological and psychological stress in athletes can affect respiration and inspiratory depth negatively [50]. A breathing that is shallow may over time evolve into thoracic breathing and become the habitual way of breathing as frequently seen in adolescent athletes presenting with dysfunctional breathing and EILO [15, 22]. Consequently, changing respiratory restrictions and habitual patterns of breathing takes time, the length of the intervention in this study was therefore five months, a length considered necessary for altering a person´s physical and emotional habitual reactions and for the individual to integrate these changes [52].

### Clinical implications

The findings in the present study suggest that treatments of inspiratory distress and EILO should:

- Be based on an interview addressing subjective experiences of respiratory distress and a thorough physical examination to identify bodily restrictions in general and breathing constraints in particular.
- The breathing pattern should be addressed. If this is thoracic, diaphragmatic breathing should be facilitated, given that such breathing is ideal for retaining an optimal position of the larynx and laryngeal movements, and it happens automatically. Measures described in the intervention section might be useful to obtain diaphragmatic breathing.
- Postural dealignments of the neck and body as a whole should also be addressed, as these also may affect the quality of the respiration and respiratory movement.
- Help the athlete to become aware of the breathing both in rest and during exercise, and provide them with measures helping them to become more in control of the breathing.
- Challenge the athletes´ thoughts on the level of daily life stress, how this can be managed and how to lower the pressure on themselves during sport related activity and competitions.
- Apply a conservative approach in the initial investigation and management of otherwise healthy children and adolescents with symptoms of EILO. The CLE test might be useful to confirm the condition and describe anatomical abnormalities contribute to EILO.
- Involve physiotherapists possessing competence on the function of the respiratory system, postural compensations and rehabilitation issues that may offer individually designed interventions for individuals striving with respiratory distress and EILO. Other professionals may also contribute constructively, such as speech language therapists and athletic coaches.

### Strengths and limitations

To our knowledge, this is the first study applying a multidimensional approach including NPMP based physiotherapy, elements of cognitive behavioural therapy and an individualised rehabilitation plan in treating adolescent athletes with EILO. Although the number of participants was relatively small (eighteen), a key strength is the multidimensional approach and the individually tailored methodologies employed, including descriptive data from semi structured interviews and clinical body assessment, as well as physiological variables from the lung function and exercise test. It may also be a strength that both bodily and cognitive features were addressed.

The physiological methods used to measure lung function and exercise tests are valid and reliable. and were conducted by the same researcher throughout the process. The first author, who is also the physiotherapist having conducted the intervention, have followed the participants throughout the intervention period. It may be considered beneficial for the participants to have one test administrator and one therapist to relate to during the course of the intervention.

Although the CLE test has been considered valuable to verify the condition, the test was not considered necessary to verify EILO in participants before inclusion in this study. The test is time consuming and expensive, requires much resources (i.e., equipment and personnel), and athletes having conducted the test in previous work report difficulties with achieving high intensity during the test [15]. The condition of EILO was confirmed via subjective experiences of respiratory distress according to the questionnaire developed by Roksund [30] in the present study.

The lack of control group poses a threat to the internal validity and generalizability of the findings, making it challenging to assess the causal relationship between the intervention and the outcome. However, considering that participants had experienced symptoms of EILO for an average of 3.2 years prior to the intervention, it is likely that the intervention itself was the driving factor contributing to the reported change in inspiratory distress. Another weakness was the use of the questionnaire developed by Roksund [30] to determine whether symptoms were indicative of EILO, as it was not developed for research purposes. Consequently, reliability and validity studies are lacking. The questionnaire was, however, functional as a basis for the pre and post interview in which respiratory distress related to exercise was explored and has commonly been used for the same purpose at a University Hospital in Bergen, Norway. The project period took place during the pandemic (March 2020—May 2021), when organized training and testing in test laboratories were restricted and competitions also had to pause. It is unclear whether the pandemic affected the results of our study.

## Conclusion

Our results suggest that a multidimensional individually tailored intervention, including NPMP based physiotherapy, cognitive behavioural therapy elements, and a rehabilitation plan might reduce inspiratory distress and dysfunctional breathing in adolescent athletes with EILO. Our findings further indicate that EILO is related to the respiratory system and body as a whole. This highlights the importance of adopting a whole-body approach when managing EILO, focusing on addressing the pattern of breathing and postural alignment. It is suggested that a conservative approach might be useful in the initial management of EILO, and that NPMP combined with self-help measures and a rehabilitation plan is a viable option for treating EILO in otherwise healthy adolescents.

## Data Availability

The datasets concerning the body examinations generated during the current study are not publicly available due to individual privacy concerns but are available from the corresponding author on reasonable request.

## Abbreviations

BF_max_: Maximal breathing frequency
BR: Breathing reserve
CI: Confidence intervals
EIB: Exercise induced bronchial constriction
EILO: Exercise-induced laryngeal obstruction
FEV_1_: Forced expiratory volume in one secon
FVC: Forced vital capacity
HR_max_: Maximal heart rate
ILO: Inducible laryngeal obstruction
NPMP: Norwegian psychomotor physiotherapy
MVV: Maximal voluntary ventilation
RER_max_: Maximal respiratory exchange ratio
R-BOP: Relaxed breathing out practice
SD: Standard deviation
VO_2max_: Maximal oxygen uptake
VE_max_: Maximal ventilation
